# Serum after Autologous Transplantation Stimulates Proliferation and Expansion of Human Hematopoietic Progenitor Cells

**DOI:** 10.1371/journal.pone.0018012

**Published:** 2011-03-18

**Authors:** Thomas Walenda, Gudrun Bokermann, Edgar Jost, Oliver Galm, Anne Schellenberg, Carmen M. Koch, Daniela M. Piroth, Wolf Drescher, Tim H. Brümmendorf, Wolfgang Wagner

**Affiliations:** 1 Helmholtz-Institute for Biomedical Engineering, RWTH Aachen University Medical School, Aachen, Germany; 2 Department for Hematology and Oncology, RWTH Aachen University Medical School, Aachen, Germany; 3 Department for Gynecology, RWTH Aachen University Medical School, Aachen, Germany; 4 Department for Orthopedics, RWTH Aachen University Medical School, Aachen, Germany; Brigham & Women's Hospital - Harvard Medical School, United States of America

## Abstract

Regeneration after hematopoietic stem cell transplantation (HSCT) depends on enormous activation of the stem cell pool. So far, it is hardly understood how these cells are recruited into proliferation and self-renewal. In this study, we have addressed the question if systemically released factors are involved in activation of hematopoietic stem and progenitor cells (HPC) after autologous HSCT. Serum was taken from patients before chemotherapy, during neutropenia and after hematopoietic recovery. Subsequently, it was used as supplement for *in vitro* culture of CD34^+^ cord blood HPC. Serum taken under hematopoietic stress (4 to 11 days after HSCT) significantly enhanced proliferation, maintained primitive immunophenotype (CD34^+^, CD133^+^, CD45^−^) for more cell divisions and increased colony forming units (CFU) as well as the number of cobblestone area-forming cells (CAFC). The stimulatory effect decays to normal levels after hematopoietic recovery (more than 2 weeks after HSCT). Chemokine profiling revealed a decline of several growth-factors during neutropenia, including platelet-derived growth factors PDGF-AA, PDGF-AB and PDGF-BB, whereas expression of monocyte chemotactic protein-1 (MCP-1) increased. These results demonstrate that systemically released factors play an important role for stimulation of hematopoietic regeneration after autologous HSCT. This feedback mechanism opens new perspectives for *in vivo* stimulation of the stem cell pool.

## Introduction

Hematopoietic stem cell transplantation (HSCT) has evolved from a highly experimental procedure to a standard therapy for several malignant and hereditary diseases [1]. Pioneering work was done more than 50 years ago by Thomas and colleagues [2]. Since then, allogeneic and autologous transplantation settings are commonly used for reconstitution of blood formation after high-dose chemotherapy. Hematopoietic recovery is usually observed within weeks. Despite this clinical success, it is yet unclear what governs this enormous regenerative potential and activation of hematopoietic stem cells (HSC) in their niche [3].

Over the last decades, umbilical cord blood (CB) has become a viable option for HSC transplants [4]. Especially for CB, the amount of transplantable HSC is limited by the available volume – therefore *in vitro* expansion might provide new perspectives for HSCT. Several growth factors have been shown to be relevant for stimulation of proliferation and maintenance of primitive function under *in vitro* conditions [5]–[8]. Cellular support such as mesenchymal stromal cells (MSC) can further enhance expansion of hematopoietic progenitor cells (HPC) [9]–[11]. Recently, the aryl hydrocarbon receptor antagonist StemRegenin 1 (SR1) has been shown to promote *in vitro* expansion of HSC [12]. Several of these approaches are currently addressed in clinical trials, but at the moment no proof exists that these expansion techniques improve performance after HSCT.

The most essential mechanism for hematopoietic recovery after transplantation is activation of the stem cell pool. These cells are defined by the dual ability to self-renew and to differentiate into distinct cell types, whereas they reside in a quiescent state under steady state conditions [13], [14]. Mathematical modeling indicated that it is more effective to increase the self-renewal rate than the proliferation rate in the course of autologous HSC transplantation and this should be mediated by a feedback mechanism [15]. A better understanding of these mechanisms might facilitate more reliable and faster hematopoietic recovery without the need of higher HSC numbers.

So far, research has mainly focused on characterization of HSC and methods for their *in vitro* expansion [16]. In contrast, methods for activation of stem cell function in the course of HSCT have hardly been addressed. The highest activation of self-renewal might be anticipated during neutropenia following high-dose chemotherapy. Under these conditions, the hematopoietic system is under an enormous regenerative pressure, and this might be regulated by systemically released feedback signals. Therefore, we have taken serum samples from patients in the course of autologous HSCT to evaluate their effect on proliferation and maintenance of primitive function of hematopoietic progenitor cells.

## Results

### Proliferation of HPC is stimulated by serum after HSCT

**Figure 1:**
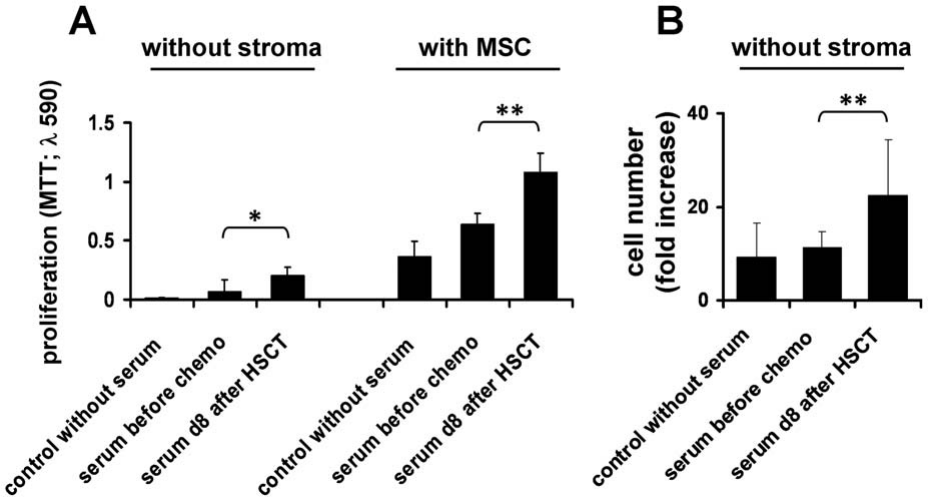
Serum after HSCT stimulates proliferation of CD34^+^ cells. Serum samples were taken before chemotherapy and eight days after autologous HSCT, when leukocyte counts are at a minimum. Serum was subsequently supplemented for *in vitro* expansion of CD34^+^ cells for 1 week either without stromal support or with co-culture of mesenchymal stromal cells (MSC). Analysis was either performed with the MTT-assay (A) or using a cell counter to determine fold-expansion rates (B). Serum from neutropenic patients significantly increased proliferation of HPC (* p<0.05; ** p<0.01; error bars represent SD).

Fifty-one serum samples were harvested from nine patients before and after HSCT (table 1). Culture medium was supplemented with 10% of these serum samples for subsequent *in vitro* expansion of CD34^+^ cells from umbilical cord blood. Proliferation of HPC was analyzed after one week by MTT assay (figure 1). Serum samples which were taken 8 days after HSCT (d8 serum; during neutropenia) significantly enhanced proliferation of HPC in comparison to those taken before chemotherapy (BC serum; experiments were repeated with serum samples of 7 patients, p = 0.03). This growth-promoting effect was also observed in co-culture with mesenchymal stromal cells (MSC; p = 0.0017). Direct counting of cell numbers revealed a 1.97-fold higher proliferation of HPC with d8 serum as compared to BC serum under culture conditions without MSC (experiments were repeated with serum samples of four patients, p = 0.0081). Overall, the results clearly demonstrate that serum taken during hematopoietic stress after HSCT significantly enhances proliferation of HPC *in vitro*.

**Table 1:** Serum samples for culture expansion of HPC.

Patient	Age	Gender	Diagnosis	Therapy	Neutropenia <500 per µl (days after HSCT)	Thrombopenia, <30,000 per µl (days after HSCT)	Serum Samples (days after HSCT)
# 1	21	f	NHL	BEAM*	1 to 8	2 to 12	BC, −1, 4, 8, 14, 22
# 2	60	m	NHL	BEAM*	1 to 7	3 to 10	BC, −1, 4, 8,14
# 3	58	f	MM	Melphalan*	5 to 9	8 to 11	BC, 1, 4, 8, 11, 14, 18
# 4	65	m	MM	Melphalan*	5 to 10	6 to 11	BC, 1, 4, 8, 11
# 5	55	m	MM	Melphalan*	5 to 10	6 to 14	BC, 1, 4, 8, 11
# 6	32	m	NHL	BEAM*	2 to 9	4 to 9	BC, 1, 4, 6, 8, 11
# 7	64	f	MM	Melphalan*	5 to 9	7 to 9	BC, 1, 4, 8, 11
# 8	61	f	MM	Melphalan	6 to 15	7 to 13	BC, 1, 4, 8, 11, 14, 18
# 9	61	m	MM	Melphalan	6 to 13	9 to 18	BC, 4, 8, 11, 14

MM =  multiple myeloma; NHL  =  non-Hodgkin lymphoma; melphalan  = 200 mg/m^2^ on d-2; BEAM  =  carmustine 300 mg/m^2^ d-7, etoposide 150 mg/m^2^ d-7 to -4, cytosine arabinoside 200 mg/m^2^ d-7 to -4 and melphalan 140 mg/m^2^ d-3;

* =  G-CSF stimulation from d0 until end of neutropenia; BC  =  before chemotherapy.

### Maintenance of CD34 expression in HPC is enhanced by serum after HSCT

**Figure 2:**
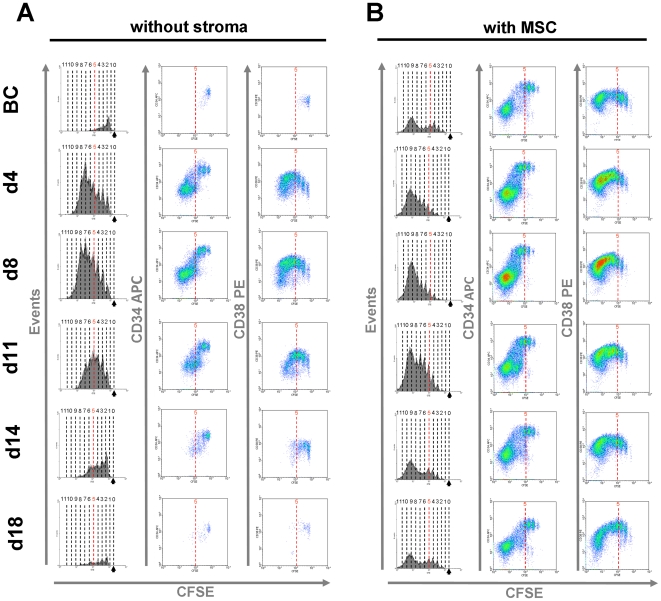
Proliferation and immunophenotype of HPC upon expansion with serum samples. CD34^+^ hematopoietic progenitor cells (HPC) were stained with CFSE and subsequently cultivated for seven days *in vitro* either without stromal support (A) or with MSC (B). Culture medium was supplemented with serum samples taken before chemotherapy (BC) or at different time points after HSCT (d4, d8, d11, d14 and d18). Flow cytometric analysis was used to monitor cell division history by residual CFSE staining (red line indicates five cell divisions). CD34 and CD38 expression was analyzed in relation to the number of cell divisions. Representative histograms for analysis of all nine patients are demonstrated.

For simultaneous analysis of cell divisions and immunophenotype, we have stained freshly isolated CD34^+^ cells from cord blood with carboxyfluorescein diacetate *N*-succinimidyl ester (CFSE). After seven days, residual CFSE dye was analyzed together with immunophenotypic markers (figure 2). Proliferation of HPC was always markedly increased with serum supplements taken 4 or 8 days after HSCT in comparison to serum isolated before chemotherapy. In contrast, no increase in proliferation was observed with serum taken 18 days or 22 days after HSCT. Co-culture with MSC greatly enhanced survival and proliferation of HPC *in vitro* and facilitated up to 12 cell divisions. Proliferation under these co-culture conditions was further increased by addition of serum samples which were taken 4 and 8 days after HSCT.

Notably, expression of primitive surface markers was also maintained for more cell divisions by addition of serum taken under hematopoietic stress: CD34 expression level was higher, and it was maintained for more than five cell divisions by supplementation of serum derived from 4 days or 8 days after HSCT. Furthermore, up-regulation and decay of CD38 expression were shifted to higher numbers of cell division. These effects were consistent in all experiments and with all patient samples used in this study. Taken together, addition of serum which was taken during neutropenia significantly enhances proliferation of HPC and maintains expression of CD34 for more cell divisions.

### Serum after HSCT enhances expansion of colony forming units

**Figure 3:**
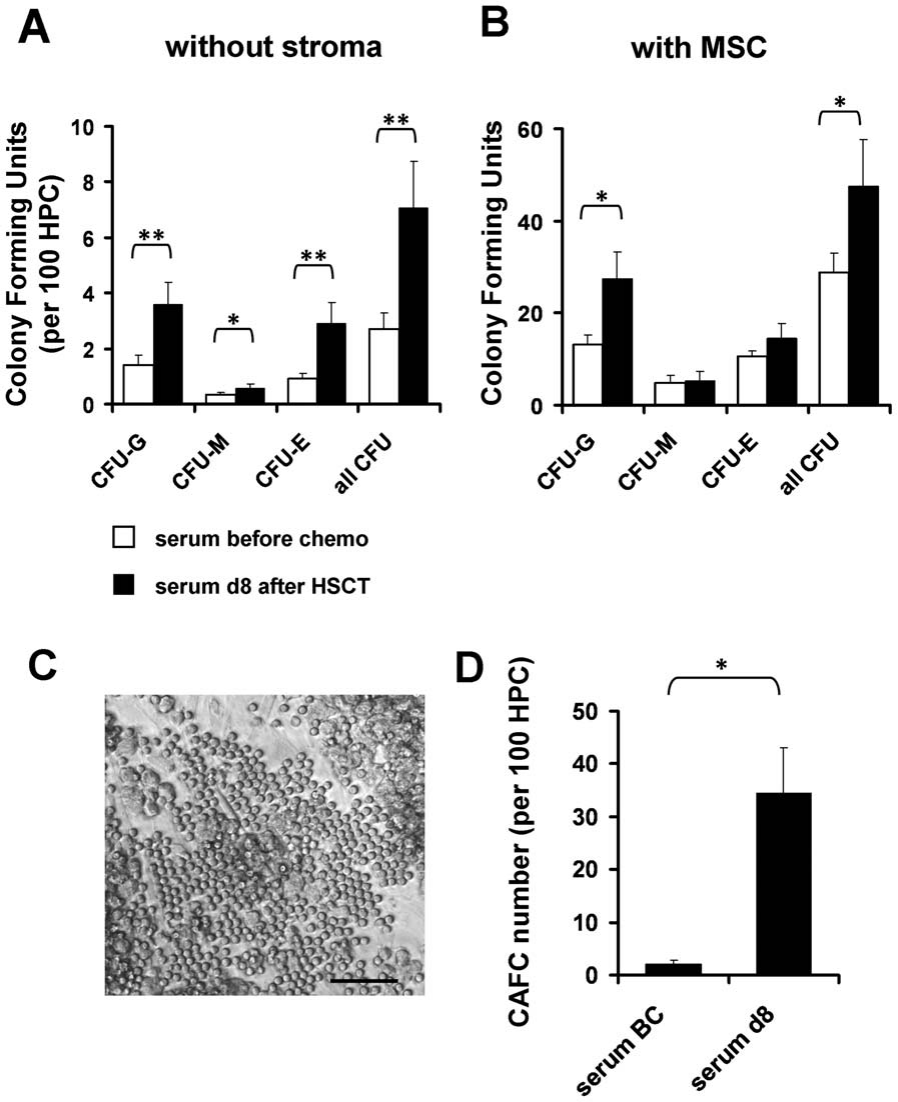
Colony forming unit frequency is enhanced by serum of aplastic patients. CD34^+^ HPC were cultured either without stroma (A) or in co-culture with MSC (B) in culture medium supplemented with serum taken before chemotherapy or day 8 after HSCT. After seven days of *in vitro* expansion, cells were re-seeded in methylcellulose medium and after two weeks numbers of granulocyte (CFU-G), macrophage (CFU-M) and erythrocyte colonies (CFU-E) were counted. Overall, colony formation was enhanced upon expansion with d8 serum. CFU-frequency was greatly increased by co-culture with MSC, and this was further stimulated by serum from d8. Furthermore, cobblestone area forming cell (CAFC) frequency was determined after 4 weeks of co-culture with MSC (C). The CAFC frequency was greatly enhanced by addition of d8 serum (D) (n = 4; *  = p<0.05; **  = p<0.01; error bars represent SEM; size bar  = 100 µm).

Subsequently, we determined colony forming unit (CFU) potential to estimate expansion of HPC. CD34^+^ cells were cultured for seven days with serum taken either before chemotherapy or eight days after HSCT (d8). Day 8 serum significantly increased CFU frequency of granulocyte (CFU-G; p = 0.001), macrophage (CFU-M; p = 0.046) and erythrocyte lineage (CFU-E; p = 0.009; figure 3). There was no bias for specific lineage specification. The number of colonies was about tenfold higher after culture-expansion with MSC, and even under co-culture conditions, expansion of CFU was significantly increased by addition of d8 serum (p = 0.039).

Alternatively, we have analyzed the frequency of cobblestone area-forming cells (CAFC) at day 28 to estimate the fraction of more primitive progenitor cells. In culture medium supplemented with d8 serum, the CAFC frequency was about 16-fold higher as compared to culture conditions with serum BC (p = 0.0358). These data provide further evidence that serum samples of neutropenic patients contain factors which enhance expansion of HPC *in vitro*.

### Up-regulation of lineage-markers

**Figure 4:**
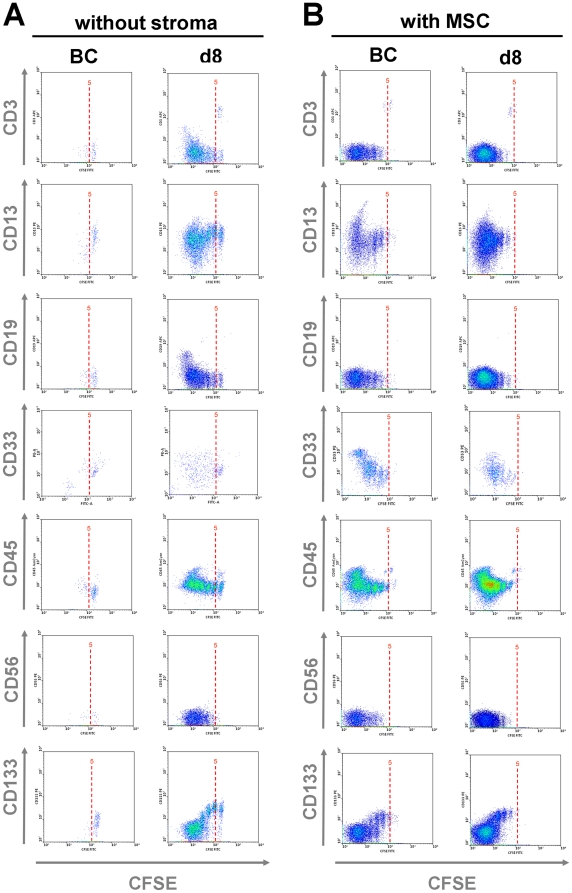
Analysis of lineage markers. CD34^+^ HPC were stained with CFSE and cultured either without stroma (A) or in co-culture with MSC (B) in culture medium supplemented either with 10% BC serum or 10% d8 serum. After seven days expression of CD3, CD13, CD19, CD33, CD45, CD56 and CD133 were analyzed by flow cytometry. Representative results for four patients are demonstrated (each of them performed in duplicate).

Culture conditions might stimulate differentiation of HPC towards specific lineages. As described above, CD34^+^ cells hardly proliferated if cultured with 10% BC serum, and they hardly express specific lineage markers (figure 4, figure S1). Expansion with d8 serum showed a moderate up-regulation of CD3 and CD19 in the fast proliferating (CFSE low) fraction and this might indicate a starting differentiation towards lymphoid lineage. Notably, up-regulation of these lymphatic markers seems to be suppressed in co-culture with MSC, whereas co-culture with MSC resulted in a moderate increase of the myeloid markers CD13 and CD33 after eight cell divisions. This is in line with other studies indicating that differentiation of cells cultured on human MSC primarily shifts toward myeloid lineage [17], [18]. The common leukocyte antigen CD45 was always up-regulated after about eight cell divisions, whereas no positive staining was observed for the NK cell marker CD56. Expression of CD133 decayed after several cell divisions in accordance to CD34. Notably, expression of this marker for primitive HPC (prominin 1) was also maintained for more cell divisions if cultured with d8 serum. These results support the notion that serum of neutropenic patients enhances proliferation of HPC and maintains their primitive phenotype for more cell divisions.

### Chemokine profiling of serum samples

**Figure 5:**
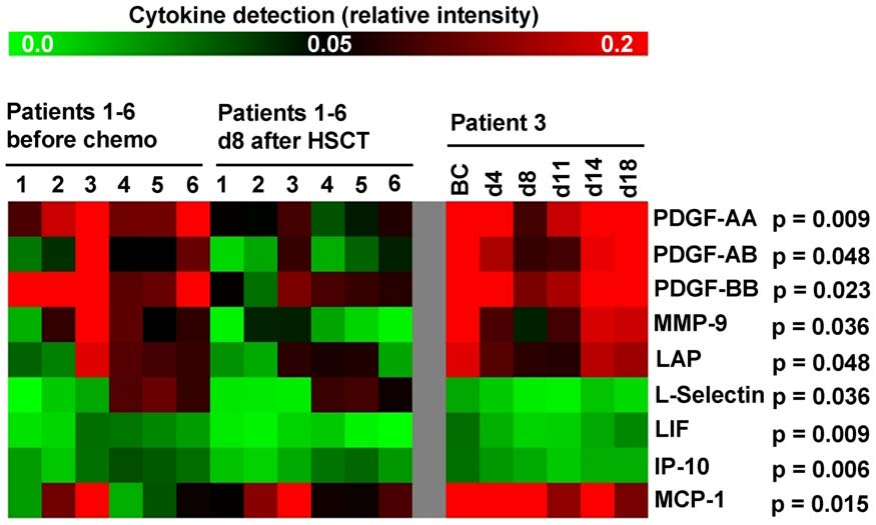
Chemokine profiling in the course of HSCT. 174 human serum proteins were analyzed in serum samples of six patients (#1–6) obtained before chemotherapy (BC) or at day 8 after HSCT. Detection of platelet-derived growth factor (PDGF)-AA, -AB, -BB, matrix metallopeptidase -9 (MMP-9), leukocyte alkaline phosphatase (LAP), L-Selectin, leukemia inhibitory factor (LIF) and chemokine [C-X-C motif] ligand 10 (IP-10) was higher before chemotherapy, whereas only chemokine [C-C motif] ligand 2 (MCP-1) significantly increased after chemotherapy. Additional comparison of chemokine profiles in serum samples at further time points (BC and from d4, d8, d11, d14 and d18 after HSCT) revealed that these changes in growth-factor concentration are transient during aplasia and normalize after hematopoietic recovery.

To determine which growth factors contribute to enhanced hematopoietic regeneration after autologous HSCT, we used Human Cytokine Antibody Arrays for analysis of 174 chemokines (figure S2). Chemokine profiles of six patients were compared in serum samples before chemotherapy and day 8 after HSCT (figure 5). Unexpectedly, pair wise comparisons revealed significant down-regulation of eight proteins in d8 serum: platelet-derived growth factor (PDGF)-AA (p = 0.009), PDGF-AB (p = 0.048), PDGF-BB (p = 0.023), matrix metallopeptidase-9 (MMP-9; p = 0.036), leukocyte alkaline phosphatase (LAP; p = 0.048), L-Selectin (p = 0.036), leukemia inhibitory factor (LIF; p = 0.009) and chemokine [C-X-C motif] ligand 10 (CXCL10 or IP-10; p = 0.006). The only moderate increase was observed for monocyte chemotactic protein-1 (MCP-1; p = 0.048). Furthermore, we analyzed one patient's serum samples at additional time points (patient 3: BC, d4, d8, d11, d14 and d18 after HSCT). This kinetic revealed that expression of these chemokines decayed during neutropenia and returned to pre-chemotherapy level 18 days after HSCT (figure 5).

Down-regulation of PDGF in day 8 serum samples might be associated with lower platelet counts and this could also contribute to the feedback signal which activates hematopoiesis. We have addressed the effect of PDGF in our culture system using serum-free conditions supplemented with 20 ng/mL TPO, 10 ng/mL FGF, 10 ng/mL SCF together with different concentrations of PDGF (0, 1, 10 and 100 ng/mL; figure S3). PDGF did not have any effect on proliferation of HPC without stromal support. In co-culture with MSC, addition of 100 ng/mL PDGF slightly impaired proliferation of HPC. Correspondingly, the relative CFSE intensity increased from 12.39 to 21.44 (p = 0.03; n = 7; figure S3). However, PDGF did not affect CD34 expression, and there was no consistent effect on colony formation either without or with co-culture. Alternatively, we analyzed if up-regulation of MCP-1 was responsible for the stimulatory effect on HPC. CD34^+^ cells were cultured in medium either with 10% patient serum BC or with TPO, FGF and SCF as mentioned above. These media were supplemented with different concentrations of MCP-1 (0, 1, 10 and 100 ng/ml). Overall, MCP-1 did not have significant impact on proliferation or CD34 expression (figure S4).

## Discussion

The present study provides evidence that systemically released factors mediate the enormous regeneration after chemotherapy-induced myelosuppression. Under steady state conditions, HSC reside in a dormant state within their stem cell niche. In contrast, hematopoietic stress recruits HSC into proliferation [13]. In HSCT, the progeny of about 2×10^8^ CD34^+^ cells gives rise to more than 3×10^10^ leucocytes in the peripheral blood. Hence, there has to be a more than hundred-fold increase in cell numbers within two to three weeks [15]. It has been shown that higher numbers of CD34^+^ cells in the transplant enhance hematopoietic recovery [19]–[21]. On the other hand, stimulation of stem cell function after transplantation appears to be at least equally important.

Expansion of HSC requires both: proliferation to increase cell numbers and maintenance of their primitive phenotype and function. This has often been considered as oxymoron: proliferation under *in vitro* conditions usually results in loss of “stemness”. Many authors have shown that expression of CD34 decays after several cell divisions by using the CFSE-dilution method [7], [22], [23]. Notably, loss of CD34 expression and the peak of CD38 expression shifted to higher numbers of cell divisions with serum at day 8 after HSCT. Furthermore, these serum samples significantly enhanced CFU frequency and CAFC frequency. It has to be noted that seven out of nine patients received G-CSF to accelerate hematopoietic recovery after HSCT and this chemokine might affect these results. However, the very same effect was also observed in the other two patients without G-CSF as well as with serum samples which were taken after chemotherapy but before the first G-CSF application. Furthermore, G-CSF serum levels seemed to be very low at the time of blood sampling as it was not detected by cytokine profiling. Therefore, the stimulatory effect of serum after autologous transplantation has to be attributed to other activating molecules.

The stem cell niche in the bone marrow is composed of multiple different cell types and hematopoiesis is probably regulated by an orchestra of different growth factors, extracellular matrix proteins, oxygen pressure and adhesion proteins [14], [24]. Co-culture experiments with MSC demonstrated that cell-cell contact with stromal cells greatly enhances proliferation and maintenance of primitive markers. Various adhesion proteins have been shown to be involved in this process [9], [11], [25]–[27]. This is complemented by a multitude of studies that demonstrated the importance of growth factors for *in vitro* culture of HSC – especially of cytokine mixtures such as SCF, TPO and FGF [5], [28], [29]. Recently, Himburg and co-workers [30] demonstrated that systemic administration of pleiotrophin (PTN) causes a substantial increase in the regeneration of HSC *in vivo* after total body irradiation of mice. Thus, better insight into the underlying mechanisms of hematopoietic regeneration might increase recovery rates.

So far, only few studies have analyzed secretion of growth factors after chemotherapy. It has been shown that TPO levels increase one week after intensive chemotherapy and decline after three weeks, indicating that TPO plays a critical role for reconstitution of megakaryopoiesis and platelet production following HSCT [31], [32]. Other authors demonstrated that interleukin-6 (IL-6) levels rise in patients with chemotherapy-induced myelosuppression [32], [33]. We did not observe significant up-regulation of TPO, and there was only a moderate increase of IL-6 after HSCT (p = 0.09). Unexpectedly, the most significant changes upon chemotherapy induced myelosuppression were down-regulation of several growth factors. Chemokines work in concert, and it is also conceivable that some of them exert negative effects on hematopoiesis – their down-regulation might correspondingly stimulate self-renewal of HSC [34], [35]. For example, it has been shown that IP-10 suppresses colony formation of HPC in a dose dependent manner [36]. This is in line with our observation that IP-10 levels decrease 8 days after HSCT. The significant decrease observed for the three isoforms of PDGF might result from lower platelet counts after chemotherapy. Other authors have indicated that PDGF is involved in expansion of HPC, but its role is not yet fully understood [37]. Recently, Aghideh et al [38] provided evidence that platelet derived growth factors suppress the expansion of CD133^+^ HPC. We observed an inhibitory effect of PDGF on proliferation of HPC only in co-culture with MSC. This effect might also result indirectly from activating MSC. Overall, it is yet unclear if the hematopoiesis supportive effect can be attributed to chemokines or rather to other molecules [39]. Metabolites, hormones or small membrane vesicles might also be relevant for the stimulatory effect of serum samples, and this will be addressed in the future.

### Conclusion

Reliable protocols for *in vitro* expansion of HSC are often considered as Holy Grail for stem cell research [40]. However, it might be much more effective to enhance hematopoietic recovery by activation of the stem cell pool *in vivo*. Our results provide evidence that this is at least partly mediated by systemically released factors. Proliferation, maintenance of primitive immunophenotype and CFU frequency were significantly enhanced by addition of serum samples taken during hematopoietic stress after HSCT. Identification of molecules that recruit HSC into action might provide new perspectives to increase recovery after chemotherapy.

## Materials and Methods

### Serum samples in the course of autologous HSCT

Ten milliliter peripheral blood were collected from patients with multiple myeloma (MM) or non-Hodgkin lymphoma (NHL; table 1) after written consent using guidelines specifically approved by the Ethic Committee of RWTH Aachen University (Permit Number: EK155/09). High dose chemotherapy preceding the stem cell rescue for patients suffering from MM was performed with high dose melphalan (200 mg/m^2^ on day -2). NHL patients received BEAM chemotherapy (BCNU 300 mg/m^2^ d-7, etoposide 150 mg/m^2^ d-7 to d-4, cytosine arabinoside 200 mg/m^2^ d-7 to d-4 and melphalan 140 mg/m^2^ d-3). At day 0 all patients were transplanted with a mean number of CD34^+^ cells of 5.0×10^6^ per kg body weight (ranging from 2.8 to 10.6×10^6^). Blood samples were collected at different days in the course of HSCT - before chemotherapy (BC), one day before HSCT (d-1) and at different time points after transplantation (d1, d4, d8, d11, d14, d18 and d22). Blood samples were transferred into a 15 mL tube (Greiner, Kremsmünster, Austria), agitated horizontally at 37°C for 1 h to allow coagulation, then incubated upright at 4°C for 4 h and finally centrifuged at 840× g for 15 min [41]. Supernatant was aliquoted and stored at −80°C until use.

### Isolation of hematopoietic progenitor cells

CD34^+^ cells were isolated from fresh umbilical cord blood after written consent using guidelines specifically approved by the Ethic Committee of RWTH Aachen University (Permit Number: EK187/08). Mononuclear cells (MNC) were separated by density gradient centrifugation on Biocoll (Biochrom KG, Berlin, Germany) and CD34^+^ cells were enriched using the human CD34 Micro Bead Kit on a MiniMACS system according to the manufacturer's instructions (Miltenyi Biotec GmbH, Bergisch-Gladbach, Germany) as described before [9].

### Isolation of mesenchymal stromal cells from human bone marrow

MSC were isolated from the *caput femoris* after the patient's written consent using guidelines specifically approved by the Ethic Committee of RWTH Aachen University (Permit Number: EK128/09). Bone fragments were flushed with PBS and MNC were subsequently isolated by density gradient centrifugation on Biocoll (Biochrom KG). MSC were seeded in 10 ng/mL fibronectin (Sigma-Aldrich, Steinheim, Germany) coated tissue culture flasks (Nunc, Roskilde, Denmark) and cultivated in Dulbecco's Modified Eagles Medium-Low Glucose (DMEM-LG, PAA, Pasching, Austria) supplemented with 2 mM L-glutamine (Sigma), 100 U/mL penicillin/streptomycin (pen/strep; Lonza, Basel, Switzerland), 10% pooled human platelet lysate (pHPL) and 2 U/mL heparin to avoid gelatinization as described before [42]. Culture medium was changed twice per week. When reaching 80% confluence, MSC were trypsinized, counted by a Neubauer counting chamber (Brand, Wertheim, Germany), and re-seeded at 10^4^ cells per cm^2^ for further expansion. For co-culture experiments we have used MSC of passage 3 to 6 (10–15 population doublings).

### Culture conditions and expansion of HPC

HPC were expanded in 24-well plates (Nunc) in StemSpan serum free expansion medium (Stem Cell Technologies, Grenoble, France) supplemented with 10% patient serum. Culture was either performed without stromal support, or CD34^+^ cells were seeded on a confluent layer of MSC. In some experiments we used serum-free medium consisting of StemSpan supplemented with 10 µg/mL heparin (Roche GmbH, Mannheim, Germany), 20 ng/mL trombopoietin (TPO; PeproTech GmbH, Hamburg, Germany), 10 ng/mL stem cell factor (SCF; PeproTech), 10 ng/mL fibroblast growth factor 1 (FGF; PeproTech) and platelet derived growth factor (PDGF; R&D Systems, Minneapolis, MN, USA) in concentrations as indicated in the text.

### Proliferation assay

Proliferation of CD34^+^ HPC under the influence of different patient sera was assessed using the Thiazolyl Blue Tetrazolium Bromide (MTT) assay [43]. 5,000 cells per cm^2^ were expanded in parallel with media supplemented with 10% of different serum samples. For co-culture experiments, we have used a layer of 30,000 MSC per cm^2^ after irradiation (20 Gy, IBL-437; Cis Bio International, Bagnols, France). After seven days of cultivation, cells were harvested by vigorous pipetting and transferred into 5 mL tubes (Becton Dickinson, San Jose, CA, USA). The samples were centrifuged 10 min at 500× g for pelleting of cells, the supernatant was discarded and samples were incubated with 1 mM MTT over night at 37°C. The samples were then centrifuged 10 min at 2,360× g for pelleting of MTT crystals. After discarding of supernatant, crystals were resolved in 150 µl 4 mM HCl in isopropanol, transferred into a 96-well plate, and optical density (OD) was measured at 590 nm with a Tecan Infinite 200 plate reader (Tecan Trading, Männedorf, Switzerland). Irradiated MSC without HPC were measured in parallel for background normalization in co-culture experiments. Alternatively, we have seeded 10,000 HPC under different culture conditions and counted their progeny after seven days with a CASY cell counter (Schärfe System, Reutlingen, Germany) within a diameter range of 7 to 20 µm.

### Analysis of cell division history

Freshly isolated CD34^+^ cells were labeled with carboxyfluorescein diacetate N-succinimidyl ester (CFSE; Sigma-Aldrich) to monitor cell divisions [9]. In brief, cells were washed in PBS and then stained with CFSE at a final concentration of 2.5 µM in PBS with 0.1% FCS (Fetal calf serum; PAA) for 10 min at 37°C. The staining reaction was stopped with ice cold RPMI (PAA) with 10% FCS for 5 min on ice followed by three washing steps with PBS. HPC were then culture expanded and CFSE is equally distributed to the two daughter cells with each cell division. After seven days, CFSE intensity was measured via flow cytometry together with additional immunophenotypic markers.

### Immunophenotypic analysis

Cells were washed in PBS, stained with CD3-allophycocyanin (APC; Becton Dickinson, San Jose, CA, USA [BD], clone HIT3a), CD13-phycoerythrin (PE; BD, clone WM-15), CD19-APC (BD, clone HIB19), CD45-V500 (BD, clone HI30), CD33-PE (Beckman Coulter, Krefeld, Germany, clone D3HL60.251), CD34-APC (BD, clone 8G12), CD38-PE (BD, clone HB-7) and CD56-PE (BD, clone B159) in a dilution of 1∶200 and analyzed using a FACS Canto II (BD) running FACS Diva software (BD). Further analysis was performed using WinMDI software (WinMDI 2.8; The Scripps Institute, San Diego, CA, USA). Discrimination between MSC and HPC was possible by forward scatter, side scatter, propidium iodide (PI) staining and CFSE-staining.

### Colony forming unit assay

Colony forming unit (CFU) potential was determined to estimate culture expansion of HPC. 10,000 CD34^+^ cells were grown for seven days under each culture condition and their progeny was subsequently re-seeded in different dilutions (1∶1, 1∶10 and 1∶100 dilution) in a 24-well culture dish with 500 µl methylcellulose medium per well (HSC-CFU light with EPO; Miltenyi Biotec). After two further weeks, granulocyte (CFU-G), macrophage (CFU-M) and erythrocyte colonies (CFU-E) were counted.

### Cobblestone area-forming cell assay

Cobblestone area-forming cell assays (CAFC) were performed to estimate the frequency of more primitive progenitor cells [44]. MSC were grown to confluence in flat-bottom 96-well plates and irradiated at 20 Gy (IBL-437; Cis Bio International). CD34^+^ cells were then seeded in three different dilutions (10, 100 and 1000 cells per well) in culture medium supplemented with 10% patient serum (either BC or d8 after HSCT). These cultures were maintained with weekly two-third-medium changes, and after four weeks cobble stone areas were analyzed by phase contrast using a Leica DM IL HC microscope (Leica, Wetzlar, Germany).

### Cytokine profiling

Analysis of serum cytokine composition was performed with RayBio Human Cytokine Antibody Array C Series 2000 (RayBiotech, Norcross, GA, USA) according to the manufacturer's instructions. Serum samples were diluted 1∶2 in blocking buffer. Horseradish peroxidase fluorescence detection was done with a LAS-3000 reader (Fujifilm Europe, Düsseldorf, Germany) and quantified using the Multi Gauge program. For normalization, each sample data value was background-subtracted and normalized by the median of positive controls represented on the array. The profiles were further analyzed using the microarray tool TIGR MeV v4.4 (Dana-Farber Cancer Institute, Boston, MA, USA) and significant chemokines were identified by paired two-sided Student's T-test.

### Statistics

All results are expressed as mean ± standard deviation (SD) or ± standard error of the mean (SEM). To estimate the probability of differences we have adopted the two-sided Student's T-test. Probability value of P<0.05 denoted statistical significance.

## Supporting Information

Figure S1 **Quantitative analysis of lineage markers.** The percentage of positive cells for CD3, CD13, CD19, CD33, CD34, CD45, CD56 and CD133 was analyzed after seven days of culture with patient serum either BC or d8 after HSCT and either without or with MSC co-culture (mean and SD of six patient samples and each of them was analyzed twice; *  = p<0.05; **  = p<0.01; ***  = p<0.001).
(TIF)

Figure S2 **Heat map of all 174 human proteins represented by the Cytokines Antibody Array.**
(TIF)

Figure S3 **Effects of PDGF on** ***in vitro*** **expansion of HPC.** CD34^+^ cells were stained with CFSE and cultured for seven days in culture medium with TPO, FGF and SCF supplemented with different concentrations of PDGF. PDGF did not affect proliferation of HPC without stromal support (A), whereas addition of 100 ng/mL PDGF slowed cell division of HPC in co-culture with MSC (B; n = 4). CFU potential was not consistently affected by addition of PDGF (C, D; n = 4; error bars represent SD).
(TIF)

Figure S4 **Effects of MCP-1 on** ***in vitro*** **expansion of HPC.** CD34^+^ cells were stained with CFSE and cultured for seven days in culture medium either with 10% patient serum (BC; A) or with TPO, FGF and SCF (B). Addition of different concentrations of MCP-1 did not affect proliferation or CD34 expression of HPC.
(TIF)
