# Supplementary figures and images for: Serum after Autologous Transplantation Stimulates Proliferation and Expansion of Human Hematopoietic Progenitor Cells
Source: PLoS One. 2011 Mar 18;6(3):e18012. doi: 10.1371/journal.pone.0018012 (PMC3060918; doi:10.1371/journal.pone.0018012)

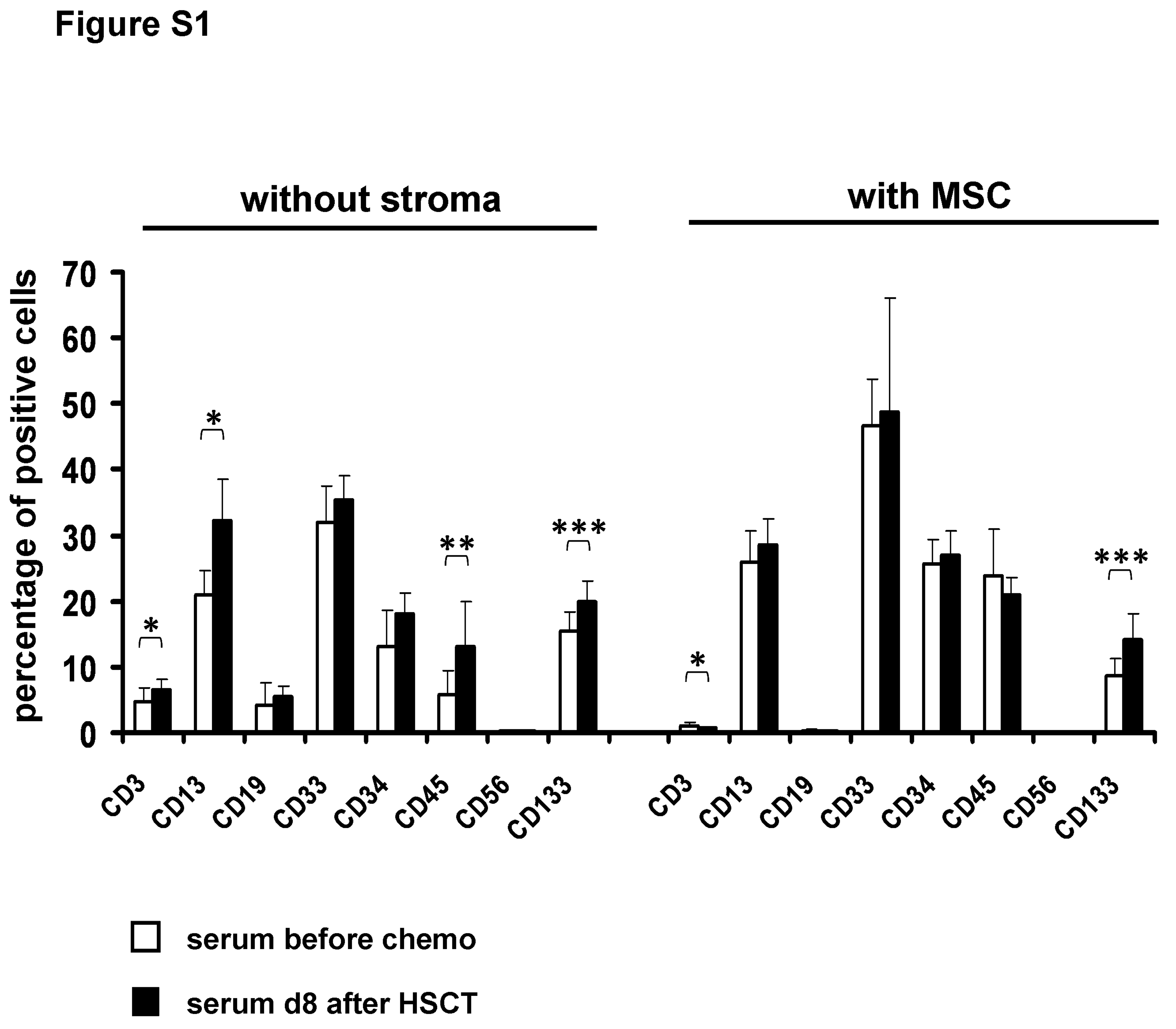

Supplement: Figure S1 — Quantitative analysis of lineage markers. The percentage of positive cells for CD3, CD13, CD19, CD33, CD34, CD45, CD56 and CD133 was analyzed after seven days of culture with patient serum either BC or d8 after HSCT and either without or with MSC co-culture (mean and SD of six patient samples and each of them was analyzed twice; * = p<0.05; ** = p<0.01; *** = p<0.001). (TIF) [file pone.0018012.s001.tif]

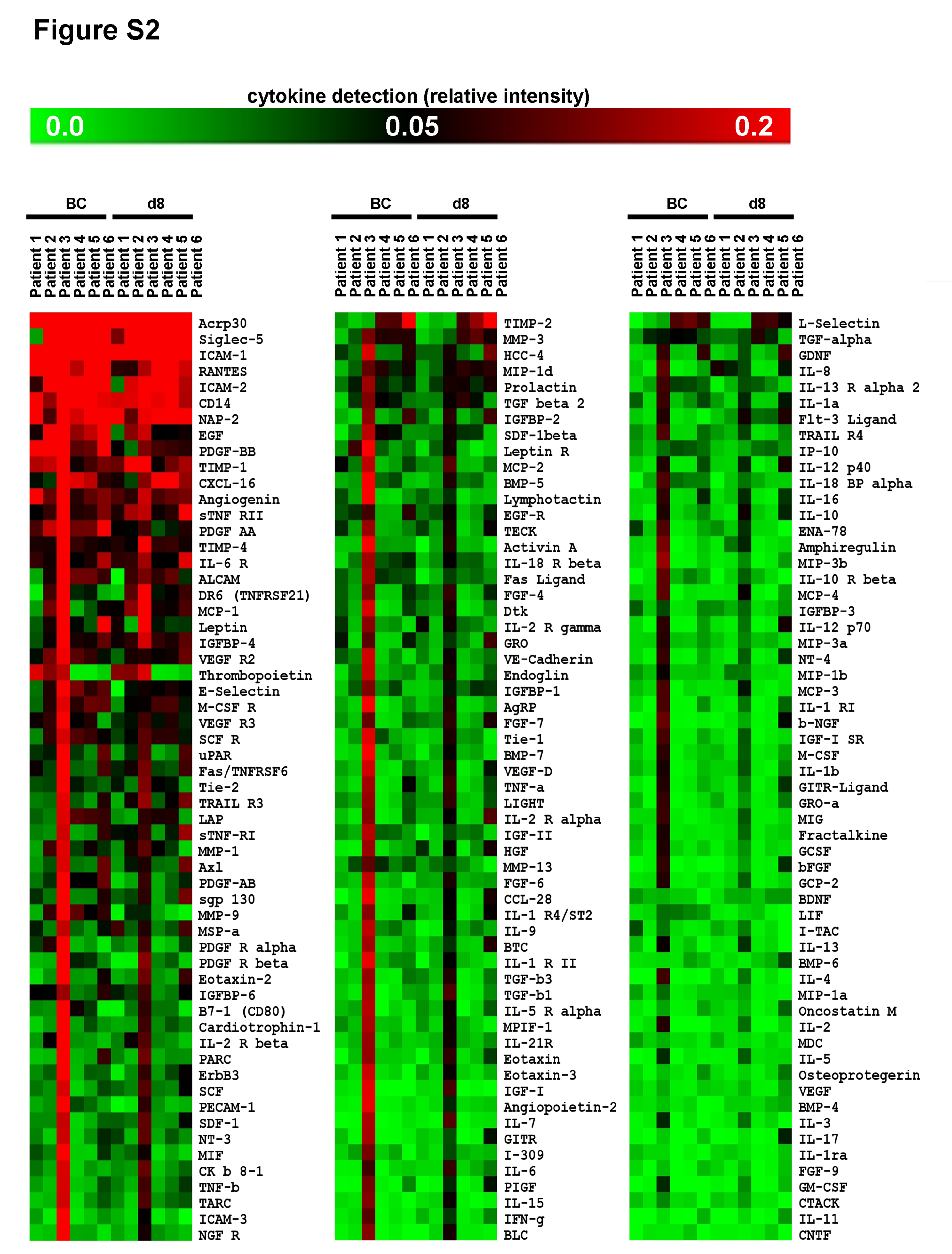

Supplement: Figure S2 — Heat map of all 174 human proteins represented by the Cytokines Antibody Array. (TIF) [file pone.0018012.s002.tif]

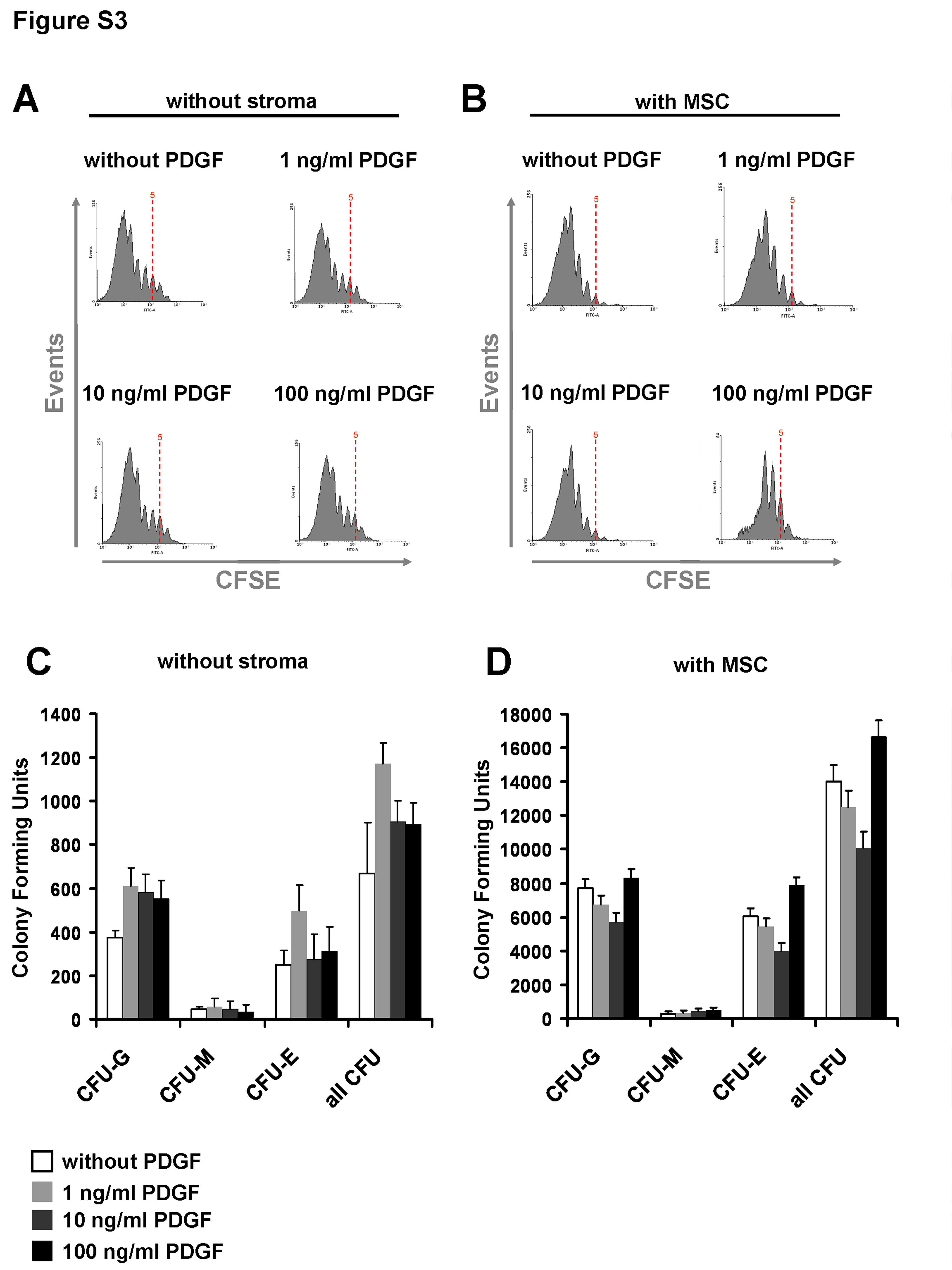

Supplement: Figure S3 — Effects of PDGF on in vitro expansion of HPC. CD34+ cells were stained with CFSE and cultured for seven days in culture medium with TPO, FGF and SCF supplemented with different concentrations of PDGF. PDGF did not affect proliferation of HPC without stromal support (A), whereas addition of 100 ng/mL PDGF slowed cell division of HPC in co-culture with MSC (B; n = 4). CFU potential was not consistently affected by addition of PDGF (C, D; n = 4; error bars represent SD). (TIF) [file pone.0018012.s003.tif]

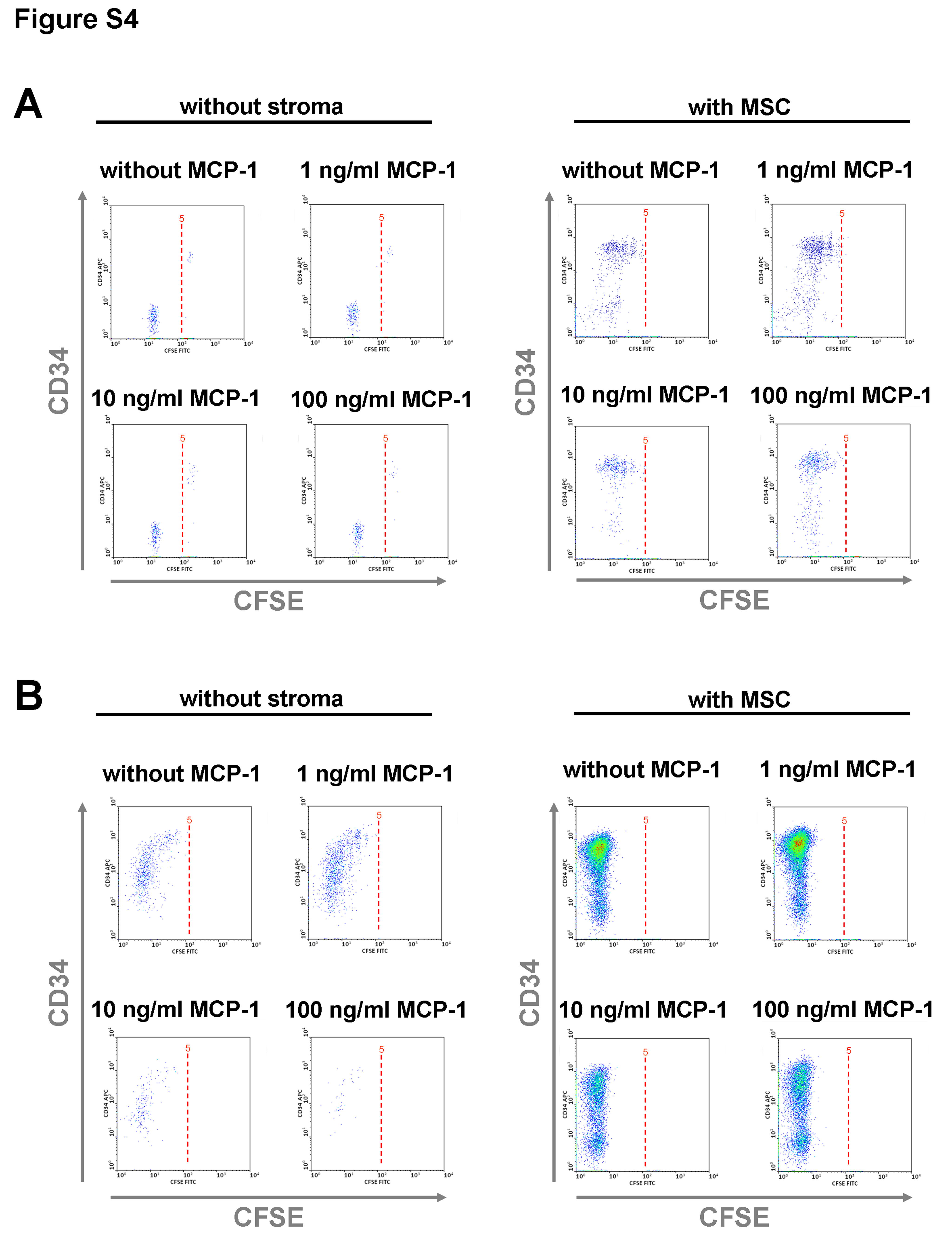

Supplement: Figure S4 — Effects of MCP-1 on in vitro expansion of HPC. CD34+ cells were stained with CFSE and cultured for seven days in culture medium either with 10% patient serum (BC; A) or with TPO, FGF and SCF (B). Addition of different concentrations of MCP-1 did not affect proliferation or CD34 expression of HPC. (TIF) [file pone.0018012.s004.tif]
